# Feasibility and acceptability of combining cognitive behavioural therapy techniques with swallowing therapy in head and neck cancer dysphagia

**DOI:** 10.1186/s12885-017-3892-2

**Published:** 2018-01-02

**Authors:** J. M. Patterson, M. Fay, C. Exley, E. McColl, M. Breckons, V. Deary

**Affiliations:** 1 Institute of Health and Society, Newcastle University, The Baddiley-Clark Building, Richardson Road, Newcastle upon Tyne, NE2 4AX UK; 2 Speech & Language Therapy Department, Sunderland Royal Hospital, Sunderland, UK; 3 Faculty of Health and Life Sciences, Northumbria University, Newcastle upon Tyne, UK; 4 Psychology Department, Northumbria University, Newcastle upon Tyne, UK

## Abstract

**Background:**

Head and neck cancer squamous cell carcinoma (HNSSC) patients report substantial rates of clinically significant depression and/or anxiety, with dysphagia being a predictor of distress and poorer quality of life. Evidence-based dysphagia interventions largely focus on the remediation of physical impairment. This feasibility study evaluates an intervention which simultaneously uses a psychological therapy approach combined with swallowing impairment rehabilitation.

**Methods:**

This prospective single cohort mixed-methods study, recruited HNSCC patients with dysphagia, from two institutions. The intervention combined Cognitive Behavioural Therapy with swallowing therapy (CB-EST), was individually tailored, for up to 10 sessions and delivered by a speech and language therapist. Primary acceptability and feasibility measures included recruitment and retention rates, data completion, intervention fidelity and the responsiveness of candidate outcome measures. Measures included a swallowing questionnaire (MDADI), EORTC-QLQH&N35, dietary restrictions scale, fatigue and function scales and the Hospital Anxiety and Depression Scale (HADS), administered pre-, post-CB-EST with three month follow-up and analysed using repeated measures ANOVA. Qualitative interviews were conducted to evaluate intervention processes.

**Results:**

A total of 30/43 (70%) eligible patients agreed to participate and 25 completed the intervention. 84% were male, mean age 59 yrs. Patients were between 1 and 60 months (median 4) post-cancer treatment. All patients had advanced stage disease, treated with surgery and radiotherapy (38%) or primary chemoradiotherapy (62%). Pre to post CB-EST data showed improvements in MDADI scores (*p* = 0.002), EORTC-QLQH&N35 (*p* = 0.006), dietary scale (*p* < 0.0001), fatigue (*p* = 0.002) but no change in function scales or HADS. Barriers to recruitment were the ability to attend regular appointments and patient suitability or openness to a psychological-based intervention.

**Conclusions:**

CB-EST is a complex and novel intervention, addressing the emotional, behavioural and cognitive components of dysphagia alongside physical impairment. Preliminary results are promising. Further research is required to evaluate efficacy and effectiveness.

## Background

Chronic swallowing difficulties (dysphagia) are a common and highly distressing side effect of surgery and/or (chemo)radiotherapy for the treatment of head and neck cancer (HNSCC) [1]. Dysphagia is associated with a higher risk of pneumonia, poor oral intake, malnutrition and prolonged tube feeding [2]. HNSSC patients report substantial rates of clinically significant depression and/or anxiety, with dysphagia being a predictor of distress [3, 4]. Our previous qualitative work reported on fundamental changes to eating habits, social lives and well-being, with some patients being better able to adjust to such changes than others [5].

Evidence based HNSCC dysphagia interventions largely centre on impairment-focused treatments delivered by speech & language therapists (SLTs) [6]. These typically include exercises to increase the range and co-ordination of swallowing function, to improve efficiency and safety. These may be administered before (as a preventative approach), during or after HNSCC treatment. The degree to which exercises prevent or reduce dysphagia is unclear due to poor patient adherence and differing exercise protocols [7]. Patients need support in coping with side effects [8], but to date, there are minimal reports of interventions addressing the psychosocial sequelae of dysphagia.

General psychosocial interventions for HNSCC patients such as psycho-education, counselling and cognitive behavioural therapy (CBT) have been reported. A Cochrane review concluded that the subsequent impact on quality of life of these interventions was uncertain due to study design limitations [9]. Whether a psychological-based treatment can be combined with impairment-based swallowing therapy to address dysphagia is an unknown. Potentially this type of intervention could improve patient engagement with rehabilitation and facilitate adjustment in living with swallowing difficulties. CBT has previously been used with HNSCC [10] and has been used by SLTs for other conditions [11].

This study aims to investigate the feasibility and acceptability of a cognitive behavioural enhanced swallowing therapy (CB-EST) for HNSCC dysphagia, using a mixed methods design.

## Methods

This is a multi-centre, prospective, longitudinal non-randomised single cohort study to explore feasibility and acceptability of a CBT enhanced swallowing therapy intervention (CB-EST) in HNSCC patients. The feasibility design was informed by guidance set out by the CONSORT 2010 statement [12].

### Patients and eligibility

HNSCC patients were recruited from two units in NE England. They were eligible for the study if they 1) had completed HNSCC treatment with curative intent 2) were medically stable and 3) scored <80 points on the MD Anderson Dysphagia Inventory [13] (swallowing specific quality of life questionnaire). Patients were excluded if they 1) had pre-existing major psychiatric diagnosis 2) had residual/recurrent HNSCC 3) were on a palliative care pathway 4) had significant communication difficulties rendering them unable to participate in a talking therapy 5) were currently receiving a psychological intervention 6) were awaiting an intervention for the purpose of improving swallowing performance (e.g. a dilatation) or 7) had significant ill-health precluding regular hospital attendance. Patients were screened and approached by members of the multi-disciplinary HNSCCC team and gave written consent before participation. The study aimed to recruit thirty participants, to provide data to perform a sample size calculation for a potential future effectiveness study [14].

### Intervention

The main researcher (JP), a SLT trained in CBT to post graduate certificate level, delivered the intervention. CB-EST was individually tailored, but aimed to include key CBT components i.e. in-depth assessment, identification of maintaining factors within a formulation, identification of a therapy goal, Socratic questioning style, cognitive and/or behavioural therapy techniques and homework tasks. The intervention also included individualised swallowing exercises, diet modifications and food texture advice, if appropriate to the patient’s therapy goal. Between 45 and 60 min were allowed for each session. Treatment was on a weekly or fortnightly basis (depending on patient preference and need for support) for up to 10 sessions by mutual agreement, with a follow up assessment at three months to monitor generalisation and maintenance. JP received supervision from an expert CBT practitioner every 2–3 weeks.

### Feasibility outcomes

As this was primarily a feasibility study, primary outcomes were those that related to the acceptability of the intervention to participants and the feasibility of trialling the intervention in a larger study. The acceptability and feasibility outcomes are as follows:

1. Acceptability was measured by the proportion of patients approached and consented and the number of sessions attended. Retention rates and reasons for drop out was documented. We aimed for a 50% recruitment rate for CB-EST to be deemed an acceptable treatment.
2. Feasibility and fidelity were measured by assessing whether the intervention could be delivered as planned, by a SLT with CBT training. Session content and treatment plans, recorded in patients’ notes were evaluated by a CBT expert practitioner as part of supervision, reliability and validity checking. Content analysis of sessions including a) whether a therapy goal was identified b) whether a CBT formulation was identified c) whether cognitive and/or behaviour change techniques were used. These outcomes would also indicate the acceptability of the intervention to patients.
3. A selection of candidate measures targeting swallowing self-report, dietary restrictions, quality of life, functioning and mood were chosen to identify appropriate tools to capture CB-EST outcomes. Acceptability to patients was monitored by percentage data completion. The measures listed below and were administered pre-, immediately following CB-EST, and at three months.

i. The MDADI [13] has twenty items, each marked using a five-point scale and summarised using a total score (range 20–100). Higher scores indicate a better outcome and a change in ≥10 points is considered a clinically significant difference [15].
ii. The European Organization for Research and Treatment of Cancer questionnaires (EORTC QLQ-C30) [16] is a general quality of life questionnaire with 30 items, five functioning scales (physical, role, emotional, cognitive, and social), three symptom scales. The EORTC QLQ-H&N35 is a disease-specific module of 35 questions divided into 7 subscales about pain, swallowing, senses, speech, social eating, social contact, and sexuality. Higher scores on the functional scales refer to better health status, whereas higher scores in symptom scales and the QLQ-H&N35 represent more severe symptoms.
iii. Chalder Fatigue Questionnaire (CFQ-11) [17] measures fatigue severity. Eleven items are answered on a four-point scale (range 0–33), with high scores representing more fatigue.
iv. Work and Social Adjustment Scale(WASA) [18] measures functional and social impairment. Five questions are answered on a nine-point scale (range 0–40) with higher scores indicating more impairment.
v. Hospital Anxiety and Depression Scale (HADS) [19] has two seven item subscales measuring anxiety (HADS-A) and depression (HADS-D). Each item is scored on a four-point scale (range 0–21 for each subscale). Subscale scores 0–7 classify participants as non-cases, 8–10 indicates borderline cases, and scores ≥11 indicate clinical levels. Total HADS scores (HADS-T) ≥ 15 indicate clinically significant distress.
vi. Performance Status Scales (PSS) Normalcy of Diet [20] measures diet texture restrictions and is clinician-rated. The scale has ten ranked categories ranging from 0 (nil by mouth) to 100 (full diet without restrictions).

The presence of a feeding tube was recorded at the same time points. The sensitivity of the candidate measures was tested by making preliminary estimates of change from pre- to post CB-EST. Data were analysed using SPSS v21 (Chicago, Illinois). We used a one way within subjects repeated measures analysis of variance complete case model. The level for statistical significance was set at 0.05. Bonferroni’s test was used for multiple post hoc comparisons. Means are reported with standard deviations and 95% confidence intervals.

4. The acceptability and feasibility of delivering CB-EST as-was or modifying it for a larger trial was further assessed using semi-structured interviews. Patients were purposively sampled to ensure a range of pre to post CB-EST changes in MDADI scores, a range of HNSCC treatment and time post-treatment. Patients were selected from those at the initial stages of CB-EST and at the end of CB-EST. Interviews were conducted by two independent researchers. Patients had the option of a telephone or face to face interview, at a time and place of their choice. All interviews were digitally recorded, transcribed verbatim and anonymised. Transcripts were read several times and in detail by the qualitative sub-team. Data were then discussed and coded using thematic analysis. Quotations relating to afore mentioned topics were independently selected and coded into key issues and themes.

### Ethics

Ethical approval was granted by the UK North East Research Ethics Committee reference 14/NE/1045.

## Results

### Feasibility and acceptability as measured by recruitment and retention

Fifty patients were screened over 20 months. Seven patients reported that their eating and drinking issues had resolved and/or they scored >80 on the MDADI, so were ineligible. Forty-three patients were approached and 30 gave written consent (69.8%). Patient characteristics for consented patients are summarised in Table 1. There was no statistical difference in distribution of gender, age, disease site, stage, type of treatment or time since treatment (*p* > 0.05) between those that consented to participation and those that did not.

**Table 1:** Patient baseline characteristics and demographics for consented CB-EST patients

Characteristics	Number (%)
Gender	
Male	26 (87)
Female	4 (13)
Age	59 (range 49–79)
Disease site	
Oropharynx	18 (60)
Oral	5 (17)
Nasopharynx	3 (10)
Larynx	2 (7)
Hypopharynx	1 (3)
Unknown primary	1 (3)
Stage	
0	1 (3)
1	2 (6)
2	13 (43)
3	7 (24)
4	7 (24)
Nodes	
0	3 (10)
1	7 (24)
2	20 (66)
Treatment	
Chemoradiotherapy	19 (63)
Surgery and radiotherapy +/− chemotherapy	9 (30)
Surgery	1 (3.5)
Radiotherapy	1 (3.5)
Time post-treatment (months)	Median 4 (IQR, Range 3,13; 1–60)
Partner	Yes (23)
	No (7)

Reasons for non-participation included difficulties with regular hospital attendance (5), did not think CB-EST would help (3), did not attend (2), currently receiving CBT (1), and too much to take on (2). Twenty five patients were retained (83%) in the intervention. Three patients dropped out at sessions 3, 4 and 5 due to disease (2 local disease, 1 lung cancer). Two patients opted not to continue with CB-EST at session 2 and 5; one found it difficult to envisage how CB-EST might be of benefit and the other felt he was making insufficient progress. No outcome data were available for drop-outs. The number of CB-EST sessions for the retained patients ranged from 3 to 10 sessions, median 6. At three month follow up, one patient was too unwell to complete questionnaires.

### Feasibility and acceptability as measured by intervention fidelity

**Fig. 1:**
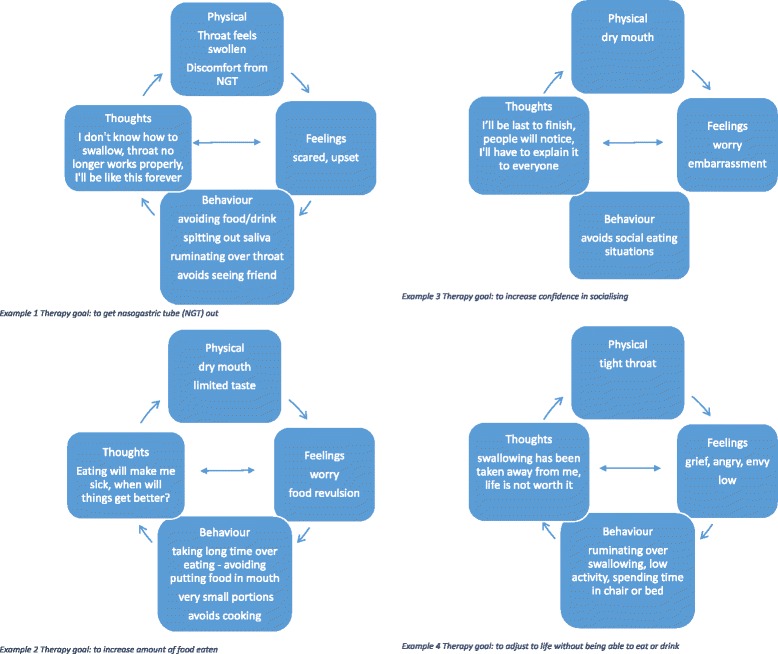
Examples of formulations from therapy goals described in Table 2

All retained patients were able to identify a goal specific to their eating and drinking problem. Goals fell into six main areas (see Table 2). A formulation for all but one drop-out patient was developed and verified during supervision. Examples of formulations are provided in Fig. 1. A range of cognitive and behavioural techniques were utilised during CB-EST and are recorded in Table 2.

**Table 2:** Therapy goals and associated CB-EST interventions for participants

	CB-EST Interventions												
Therapy goal	Swallowing exercises	Swallowing posture recommendation	Texture modification advice	Food selection/preparation advice	Size and positioning of bolus advice	Education on eating	Education on management of choking	Sleep/fatigue management	Graded behavioural experiments	Activity scheduling	Exposure/graded tasks	Thought records	Identifying unhelpful thinking habits
To get feeding tube out (*n* = 11)	x	x	x	x	x	x	x	x	x	x	x	x	x
To increase eating amount (*n* = 8)	x									x	x		x
To increase confidence in socialising (*n* = 4)									x		x	x	x
Become more confident about eating (*n* = 4)			x	x			x	x	x				x
Feel better about changes to eating and drinking (n = 2)			x	x		x	x	x		x		x	x
To adjust to life without eating and drinking (n = 1)								x	x	x		x	x

### Acceptability and utility of candidate outcome measures

Data completeness ranged from 76 to 100%, the PSS Normalcy of diet scale having the highest compliance, QLQ-C30 having the lowest compliance (see Table 3). Post CB-EST improvements were observed in several measures. Although not powered for effectiveness, a statistically significant improvement was observed in MDADI scores (*p* = 0.002, mean difference 8.1); three domains of QLQ-C30 function scales (*p* = 0.004–0.03 mean difference 10.4–21.3); seven domains on QLQ-HN35 symptoms scales (*p* < 0.0001–0.01 mean difference 10.8–14.7); CFS (p = 0.002 mean difference 4.1) and PSS Normalcy of diet scale (p < 0.0001 mean difference 15.8). These improvements were maintained at three months. No statistical improvement was observed for two domains on QLQ-C30 functioning scales; two domains on QLQ-HN35 symptom scales; WASA scales or the HADS (see Table 3).

**Table 3:** Questionnaire and feeding tube results for CB-EST

Measure	Pre-CB-EST	Post CB-EST	3 months	ANOVA, Effect size (ES)	Pre to post [95% CI]	Pre to 3 m [95% CI]	Post to 3 m [95% CI]
MDADI	*n = 25 (100%)*	*n = 23 (92%)*	*n = 24 (96%)*				
	53.1 (15.1)	61.2 (15.6)	61.8 (16.0)	*p = 0.002* (2,42) F = 8.8 ES = 0.29*	*p = 0.03* [0.7–15.5]	*p = 0.003* [2.6–14.6]	*p = 1.0* [3.6–4.7]
QLQ-C30	*n = 25 (100%)*	*n = 22 (88%)*	*n = 19 (76%)*				
Physical	63.7 (21.8)	74.1 (21.9)	74.4 (21.8)	*p = 0.006* (2,34) F = 5.9 ES = 0.26	*p = 0.05* [0.1–28.8]	*p = 0.03* [0.7–20.8]	*p = 1.0* [−8.1–7.2]
Role	48.1(38.3)	61.1(34.7)	72.2(34.3)	*p = 0.03* (1,20) F = 4.8 ES = 0.22*	*p = 0.5* [10.9–36.8]	*p = 0.06* [1.2–49.4]	*p = 0.01* [2.3–19.9]
Emotional	67.6 (31.0)	75.9 (31.7)	75.4 (24.8)	*p = 0.15* (1,17) F = 2.3 ES = 0.11	*p = 0.06* [0.4–17.1]	*p = 0.4* [5.9–21.7]	*p = 1.0* [11.9–12.9]
Cognitive	70.4 (34.6)	75.9 (34.9)	87.0 (19.4)	*p = 0.06* (2,21) F = 3.8 ES = 0.18*	*p = 0.2* [2.4–13.5]	*p = 0.1* [2.6–35.9]	*p = 0.4* [7.8–30.0]
Social	44.4 (37.0)	65.7 (36.8)	62.9 (36.8)	*p = 0.004* (2,34) F = 6.5 ES = 0.28	*p = 0.03* [1.1–41.4]	*p = 0.01* [3.8–33.2]	*p = 1.0* [12.9–18.5]
*Symptoms*							
Fatigue	40.7(24.9)	33.9 (26.8)	26.5(25.6)	*p = 0.02* (2,34) F = 4.2 ES = 0.2	*p = 0.79* [8.8–22.4]	*p = 0.02* [1.4–27.0]	*p = 0.2* [2.7–17.5]
Nausea	12.9 (25.9)	5.5 (9.9)	8.3 (17.4)	*p = 0.2* (2,34) F = 1.6 ES = 0.1	*p = 0.41* [5.1–19.9]	*p = 0.86* [−6.5–15.8]	*p = 1.0* [−11.7–6.2]
Pain	31.5 (32.3)	29.9 (34.8)	26.8 (30.3)	*p = 0.71* (2,34) F = 0.3 ES = 0.02	*p = 1.0* [14.4–25.5]	*p = 1.0* [18.5–27.7]	*p = 1.0* [12.7–14.5]
QLQ-H&N35	*n = 25 (100%)*	*n = 22 (88%)*	*n = 22 (88%)*				
Oral pain	32.2 (19.8)	23.3 (22.7)	27.1 (21.9)	*p = 0.29* (2,38) F = 1.3, ES = 0.06	*p = 0.29* [−4.0–19.8]	*p = 1.0* [−9.8–17.5]	*p = 1.0* [−17.3–9.8]
Swallow	45.8 (31.2)	35.0 (26.0)	28.3 (18.2)	*p = 0.006* (2,38) F = 5.9, ES = 0.24	*p = 0.2* [−4.1–25.7]	*p = 0.01* [3.8–31.1]	*p = 0.46* [−5.1–18.4]
Senses	45.5 (29.4)	32.5 (21.2)	22.5 (26.6)	*p < 0.0001 *(2,38) F = 10.7, ES = 0.36	*p = 0.09* [−1.8–21.2]	*p = 0.003* [6.3–33.7]	*p = 0.02* [1.4–18.6]
Speech	40.8 (35.6)	28.3 (35.9)	20.8 (22.8)	*p = 0.01* (2,38) F = 5.1, ES = 0.21	*p = 0.02* [1.1–28.9]	*p = 0.04* [0.8–39.2]	*p = 0.89* [−10.9–25.8]
Social eating	58.7 (37.8)	36.1(27.0)	38.3 (35.1)	*p = 0.006* (2,38) F = 5.9, ES = 0.24	*p = 0.03* [1.2–44.1]	*p = 0.05* [−0.5–41.4]	*p = 1.0* [−16.0–11.6]
Social context	31.0 (25.9)	16.3 (22.7)	15.7 (17.5)	*p = 0.001* (2,38) F = 9.0, ES = 0.32	*p = 0.01* [2.7–26.6]	*p = 0.004* [4.5–26.1]	*p = 1.0* [−8.6–9.9]
Sexuality	53.3 (44.1)	48.3 (46.2)	35.0 (46.1)	*p = 0.12* (2,38) F = 0.23, ES = 0.11	*p = 1.0* [−21.8–31.8]	*p = 0.06* [−0.7–37.4]	*p = 0.47* [−10.3–37.0]
CFS	*n = 25 (100%)*	*n = 24 (96%)*	*n = 22 (88%)*				
	20.3 (5.0)	16.2 (6.8)	15.9 (5.9)	*p = 0.002* (2,42) F = 7.3, ES = 0.39	*p = 0.04* [0.1–8.1]	*p = 0.04* [0.1–8.1]	*p = 1.0* [2.5–2.9]
WASA	*n = 25 (100%)*	*n = 23 (92%)*	*n = 22 (88%)*				
	20.0 (9.2)	13.9(11.6)	16.9 (22.5)	*p = 0.34*(2,40) F = 1.1, ES = 0.05*	*p = 0.07* [0.5–12.7]	*p = 1.0* [9.2–15.3]	*p = 1.0* [9.2–15.3]
HADS	*n = 25 (100%)*	*n = 23 (92%)*	*n = 22 (88%)*				
Anxiety	6.9 (4.9)	6.6 (5.9)	5.9 (4.1)	*p = 0.54* (2,38) F = 0.6, ES = 0.03	*p = 1.0* [−1.8–2.7]	*p = 0.8* [−1.4–3.3]	*p = 1.0* [−1.6–2.6]
Depression	6.0 (3.4)	5.6 (4.1)	4.9(4.1)	*p = 0.49* (2,38) F = 0.7, ES = 0.37	*p = 1.0* [−2.0–2.8]	*p = 0.78* [−1.4–3.6]	*p = 1.0* [−1.6–3.0]
Total	12.8 (7.1)	12.0 (9.6)	10.8 (3.4)	*p = 0.38* (2,38) F = 1.0, ES = 0.05	*p = 1.0* [−2.9–4.6]	*p = 0.58* [−1.9–6.0]	*p = 1.0* [−2.7–5.1]
PSS Diet	*n = 25 (100%)*	*n = 25 (100%)*	*n = 24 (96%)*				
	39.2 (19.9)	55.0 (14.4)	61.3 (18.5)	*p < 0.0001 *(2,46) F = 15.0, ES = 0.4*	*p = 0.002* [5.3–26.4]	*p = 0.001* [8.3–35.8]	*p = 0.8* [0.4–12.9]
Feeding tube	*n = 30*	*n = 25*	*n = 25*				
None	14 (56%)	22 (88%)	22 (88%)				
Nasogastric	9 (30%)	1 (4%)	0				
Gastrostomy	7 (24%)	2 (8%)	3 (12%)				

QLQ-C30, MDADI, CFS, WASA, HADS, PSS Diet higher score is better patient reported outcome. QLQ-H&N35 and HADS lower score indicates better patient reported outcome *sphercity violated for repeated measures ANOVA and adjusted using epsilon correction

### Feasibility and acceptability as measured by participant interviews

Sixteen patients were approached for interview and 15 consented. The interviewer was unable to arrange a convenient time for three patients. There were ten telephone and two face to face interviews. One patient opted to be interviewed with her partner present. The sample reflected baseline characteristics for gender, age, site and stage of disease (see Table 4).

**Table 4:** Characteristics of interview participants

Gender	
Male	9
Female	3
Age	61 (range 49–70)
Disease site	
Oropharynx	8
Oral	1
Nasopharynx	2
Larynx	1
T Stage	
2	5
3	5
4	2
Node stage	
0	2
1	3
2	7
Treatment	
Chemoradiotherapy	9
Surgery and radiotherapy +/− chemotherapy	3
Time post-treatment (months)	Median 5 range 1–60
MDADI pre to post CB-EST	
Decreased (4–19 points)	3
Similar (0–4 points)	3
Increased (5–36 points)	6

Three main CB-EST process themes were identified.

### Duration and frequency

Participants were referred to CB-EST either by a specialist nurse or a SLT and all felt this was an appropriate pathway. Overall, patients were happy with the duration of sessions. Session length of approximately one hour was deemed sufficient to explore issues and to decide on homework until the following session. Generally, patients felt that between 8 and 10 sessions was an appropriate set of meetings. Some felt that regular intervals (weekly/fortnightly) were beneficial whilst others would have preferred them to be more frequent in the early stages of CB-EST.

*‘The timeframe between that was just balanced nicely and allowed me to kind of make those plans and have an experience to then come back and talk about it, so yeah it worked well’* (S3 aged 51 yrs).

Participants understood that sessions were a finite resource and moving on was an inevitable and necessary part of the intervention. Several participants stated that they would have happily continued as they discovered an overall benefit on recovery and well-being. Patients liked the combination of talking, visualising issues using a whiteboard and other applied techniques, and concrete goal setting.

### Timing

Most participants felt that the timing of their participation in CB-EST worked well, with some expressing that they wished they had earlier access.

*‘Maybe it should be part of the initial journey and treatment … not everybody might take it but at least it is there isn’t it’ (S7 aged 61 years).*

There was variation about whether there is an ideal time point to start sessions. Timing seemed to be related to individual preferences and symptom severity. Participants seem to fall roughly into two groups: those who would prefer the sessions to accompany their treatment (i.e. start soon after diagnosis) and those who thought sessions are more beneficial following (but not too long after) treatment.

### Suitability

Some participants described themselves as being suited to an intervention such as CB-EST, i.e. either being open to talk about emotions and/or relatively open to help and change. Others described themselves as being more reluctant, not used to discussing emotional issues, and not the type to require psychological support.

*‘It will not fit everybody. Some people probably won’t want to sit there and say about their life and say how your wife was in tears and say how you were in tears and talk about things like that‘(S10 aged 54 years).*

However, even those unaccustomed to this approach felt techniques and tasks were tailored to their requirements. In part, due to the ability to tailor the intervention, all participants agreed that there is potential for anyone to benefit from CB-EST. Some said that they didn’t know what to expect but either approached sessions with an open mind or simply a ‘nothing to lose’ attitude. Even those who thought they were less likely to get much out of the sessions reported benefit in interviews, which was not always reflected in their MDADI scores.

## Discussion

CB-EST is a novel treatment and had good rates of patient uptake and retention; formulations and goals were possible for most, candidate measures had good uptake and completeness providing some evidence of effect, and patients reported that they liked the intervention. CB-EST was delivered by a CBT trained SLT and was completed within ten sessions. Results need to be interpreted with caution as patients were self-selecting and not consecutively screened and therefore it is likely that more willing patients volunteered for the intervention. Recruitment rates were marginally lower than those reported in other general HNSCC psychosocial interventions [10]. Early indications are that CB-EST is an acceptable intervention for a range of HNSCC patients not confined to treatment type, time post-treatment, or site of disease. The sample was weighted towards patients with advanced staged disease, the majority having combined modality treatment. This was likely due to the predominance of self-reported dysphagia by patients treated with chemoradiotherapy or surgery plus adjuvant radiotherapy [1, 21]. Interview data suggest that people had individual preferences as to how soon CB-EST might be offered following their treatment. Barriers to recruitment were identified. Practicalities of regular out-patient attendance needs to be taken into consideration following intensive HNSCC treatment regimens as well as additional financial costs to the patient [22]. Not all HNSCC patients wish to receive psychological support and may be unsuitable for such an intervention [23]. Drop-out rates due to disease or ill-health are expected but unavoidable in the HNSCC population.

Our previous qualitative work showed that patients view their eating and drinking problems more broadly than just physical impairment [5]. CB-EST was able to respond to individual need, addressing the psychosocial issues of dysphagia by integrating core features of CBT alongside swallowing therapy, with some reporting general improvement in their quality of life. The most common patient goal was removal of feeding tube, which involved a range of physical, cognitive and behavioural techniques. Addressing unhelpful thinking habits was a technique used across all goals. Goals for improving confidence in social eating or adjustment to permanent non-oral feeding did not require impairment based swallowing therapy.

This study employed a selection of candidate measures to assess patient acceptability and sensitivity to change. All achieved a completion rate of ≥88%. Outcomes specifically related to eating and drinking (MDADI, EORTC HN35 Swallow and Social Eating and PSS Diet score) were responsive to change, although lack of follow up data from drop outs may positively skew results. Elsewhere, a comparable longitudinal cohort study reported a MDADI mean difference of 4.7 points in the first year post-treatment [24], suggesting some spontaneous adjustment occurs. Under trial conditions for a prophylactic swallow exercise intervention, minimal change was seen in EORTC HN35 Swallow and Social Eating scores (mean difference 0 and 5) [25]. The current study found no change on the HADS, despite CBT being an effective intervention for anxiety and depression. This may be due to the sample size, although approximately two thirds of pre-CB-EST patients were either non-cases or had borderline mood issues according to cut-off criteria.

### Future work

This preliminary study directs several areas for further investigation. Future work on refining the selection criteria is required, with early results suggesting that some patients may be more suitable for CB-EST than others. CB-EST was delivered by a single SLT; whether SLTs are willing to be trained, the extent of training and access to regular supervision is unknown. In order to assess effectiveness, CB-EST would need to be protocoled, while allowing for an individually tailored approach, with fidelity checks. A proportion of patients declined participation, citing difficulties with regular attendance. It is uncertain as to whether CB-EST requires face to face intervention or if components could be administered via other mediums such as telemedicine, self-help booklets or web-based programmes. However, patients often express a preference for individual, face to face help, preferably at home [23]. It is unknown as to whether patients would be willing to be randomised in the context of a trial. Using MDADI scores, a sample size for a future trial would require 84 patients, providing 80% probability of detecting a difference at two sided significance level of 0.05. Accounting for an intervention completion rate of 58% from the available sample, 145 patients would be required to conduct such a study.

## Conclusion

The addition of cognitive behavioural techniques to swallowing therapy delivered by a trained SLT, is a feasible and acceptable treatment, addressing the physical and psychosocial components of HNSCC dysphagia. Further work is needed to establish efficacy, effectiveness and cost-effectiveness of the intervention, in the context of a randomised controlled trial.

## Abbreviations

CB-EST: Cognitive Behavioural-Enhanced Swallow Therapy
CBT: Cognitive Behaviour Therapy
CFQ: Chalder Fatigue Questionnaire
EORTC-HN: European Organization for Research and Treatment of Cancer – Head and Neck module
EORTC-QLQ: European Organization for Research and Treatment of Cancer- Quality of Life Questionnaire
HADS: Hospital Anxiety and Depression Scale
HNSCC: Head and neck squamous cell carcinoma
MDADI: MD Anderson Dysphagia Inventory
NGT: Nasogastric tube
PSS: Performance Status Scale
SLT: Speech and Language therapist
WASA: Work and Social Adjustment Scale
